# Transforming Smart Healthcare Systems with AI-Driven Edge Computing for Distributed IoMT Networks

**DOI:** 10.3390/bioengineering12111232

**Published:** 2025-11-11

**Authors:** Maram Fahaad Almufareh, Mamoona Humayun, Khalid Haseeb

**Affiliations:** 1 Department of Information System, College of Computer and Information Sciences, Jouf University, Sakaka 72388, Al-Jouf, Saudi Arabia; 2 School of Computing, Engineering and the Built Environment, University of Roehampton, London SW15 5PH, UK; 3 Department of Computer Science, Islamia College Peshawar, Peshawar 25120, Pakistan; khalid.haseeb@icp.edu.pk

**Keywords:** healthcare system, trustworthiness, cyber threats, security, smart technologies

## Abstract

The Internet of Medical Things (IoMT) with edge computing provides opportunities for the rapid growth and development of a smart healthcare system (SHM). It consists of wearable sensors, physical objects, and electronic devices that collect health data, perform local processing, and later forward it to a cloud platform for further analysis. Most existing approaches focus on diagnosing health conditions and reporting them to medical experts for personalized treatment. However, they overlook the need to provide dynamic approaches to address the unpredictable nature of the healthcare system, which relies on public infrastructure that all connected devices can access. Furthermore, the rapid processing of health data on constrained devices often leads to uneven load distribution and affects the system’s responsiveness in critical circumstances. Our research study proposes a model based on AI-driven and edge computing technologies to provide a lightweight and innovative healthcare system. It enhances the learning capabilities of the system and efficiently detects network anomalies in a distributed IoMT network, without incurring additional overhead on a bounded system. The proposed model is verified and tested through simulations using synthetic data, and the obtained results prove its efficacy in terms of energy consumption by 53%, latency by 46%, packet loss rate by 52%, network throughput by 56%, and overhead by 48% than related solutions.

## 1. Introduction

Distributed networks with cloud processing have enabled vital capabilities for real-time sensing and computing of data from the real world, as well as for communication standards [1,2]. Advancements in IoMT enable the timely collection and maintenance of patient data through wireless technologies and interconnected health devices. Such intelligent systems are nowadays more flexible and offer a diverse range of services to their networked users [3]. They integrate computational algorithms and physical components via the Internet of Things (IoT) to monitor and control complex health operations [4,5]. By fusing cutting-edge technologies and edge computing, many network applications transform the efficiency and productivity of crucial operations [6,7,8]. However, the increasing reliance on IoMT networks presents new challenges, particularly in ensuring the security of sensitive patient data and maintaining low-latency, real-time performance. The automated process, equipped with smart sensors, not only meets the application’s requirements but also reduces operational costs for controlling devices [9,10,11]. In recent decades, considerable efforts have been made by researchers to address the significant challenges in developing lightweight smart solutions that maintain the timely availability of network resources [12,13,14]. AI-based schemes are widely explored for effective decision making and timely responses to automate systems in critical environments, leading to sustainable growth for smart cities [15,16,17]. The emerging technologies in IoT-based operations demand data protection against cyber threats to maintain authorized access to sensitive health records, supported by trusted devices [18,19,20]. This research study explores AI-driven techniques to propose evaluation of trusted devices, network threats, and anomalies that are reduced, addressing cyber attacks and enabling more consistent, distributed development for emerging health technologies. The following research inquiries are addressed by our research study.

i. How can the proposed system ensure data privacy and integrity with AI-enabled techniques without affecting network resources?
ii. What methods can healthcare applications explore to decrease congestion and provide a responsive system by optimizing resources through efficient communication and network scalability?
iii. What kind of potential threats can be addressed by healthcare applications using an AI model to reduce unauthorized access and vulnerabilities to cyber attacks?

Based on the aforementioned research questions, our proposed work contributes the following developments.

i. Design and implementation of smart healthcare solution using AI-powered security for ensuring privacy of health records while affecting its integrity in a distributed environment.
ii. Edge computing is integrated for real-time processing and increases the scalability of the IoMT network while reducing congestion among health devices, and improves the responsiveness of the system in critical conditions.
iii. AI-based models are explored to ensure network availability and provide a lightweight communication paradigm for coping with network disruptions in the presence of cyber threats affecting healthcare services.

Our research work is organized in the following sub-sections. Related work is explained in the context of problem formulation in Section 2. Section 3 discusses the developed algorithms. The experiments and their results are discussed in Section 4. In conclusion, Section 5 presents the findings of this study.

## 2. Literature Review

Medical equipment with IoMT devices interacts through wearable sensors and emerging technologies to sense and process medical records in real-time environments [21,22]. The patient’s data is collected by health sensors promptly and forwarded to the nearest processing devices. Later, cloud servers perform diagnostic operations on it, enabling the rapid identification of any disease with the consultation of healthcare experts. However, in the entire process, data security is a crucial phase to ensure accurate data collection at the processing edge and prevent any alteration due to cyber threats [23,24]. The effective measurement of security levels is crucial for protecting patient privacy and ensuring reliability in a critical environment. The use of decentralized processing of health data, supported by edge-level computing, enhances the efficiency of decision making at the point of care, without relying on distant cloud servers [25,26,27]. Ref. [28] enhanced the performance of MWSNs by integrating dual-tier clustering and virtual network zones. A Dual Tier Cluster-Based Routing (DTC-BR) protocol was proposed, which divides the network into virtual zones for improved connectivity and selects specific nodes as cluster heads for optimal data transmission. The simulations were conducted in MATLAB to analyze DTC-BR and assess various network metrics, including lifetime, scalability, and energy consumption. To select the optimal phantom node and determine a secure routing path for source location privacy in an IoT-enabled blockchain healthcare, Ref. [29] proposed a novel Fractional Flamingo Archery Optimization (FFAO). It integrates various performance and multi-objective optimization parameters to enhance network security and reliability. Furthermore, an additional layer is integrated into FFAO with the support of blockchain technology, addressing factors such as distance, energy, trust, and heterogeneity, to enhance adaptability and security. The authors of [30], focusing on sustainability, introduced a software architecture to enhance the data collection process through efficient IoT-based communication. It provides QoS-aware routing and enhances the effective utilization of network resources in smart cities. During the data collection phase, Chaotic Bird Swarm Optimization (CBSO) is employed to form sensor clusters, while the Improved Differential Search (IDS) method assesses node reliability and selects the most trustworthy node as the Cluster Head. In the data transport phase, the system ensures data security through lightweight signcryption, while the Optimal Data Routing (ODM) technique enhances data transmission paths within the network. In Ref. [31], the authors presented a sink-mobility-based energy-efficient data dissemination (SEEDI) protocol for IoMT, designed to address the hot-spot problem in static wireless body area networks (WBANs). In SEEDI, four continuously energy-powered body sensor nodes are deployed at the periphery of a rectangular network to collect data from nearby patients. The sink collects data from these nodes, which in turn gather information from the patients. Cluster Head (CH) selection for each patient is performed using the Remora Optimization Algorithm (ROA), which optimizes fitness parameters to enhance energy efficiency. The expansion of the IoMT has improved the functionality, connectivity, and intelligence of medical practices. However, this increased interconnectivity has made IoMT networks vulnerable to cyber attacks, particularly Distributed Denial-of-Service (DDoS) attacks. Existing intrusion detection systems (IDSs) struggle to address these advanced threats due to the dynamic nature of IoMT traffic. The authors of Ref. [32] presented a hybrid deep-learning-based intrusion detection system (HIDS-IoMT) that combines Convolutional Neural Networks (CNNs) for feature extraction and Long Short-Term Memory (LSTM) networks for sequence prediction. The proposed IDS is implemented on a Raspberry Pi device using a fog computing architecture, enabling decentralized processing to enhance responsiveness and reduce latency. To improve the efficiency and security of patient record management, Ref. [33] proposed a framework, Healthcare System using Private Blockchain-based Cloud IoT (HSPBCI), aiming to limit access to medical data by unauthorized devices. Additionally, it enhances block-based transaction encryption and decryption times with minimal overhead. The cloud blockchain network is created to identify unauthorized devices and increase the trustworthiness of communication. Table 1 outlines the contributions and limitations of the existing approaches.

## 3. Materials and Methods

**Figure 1:**
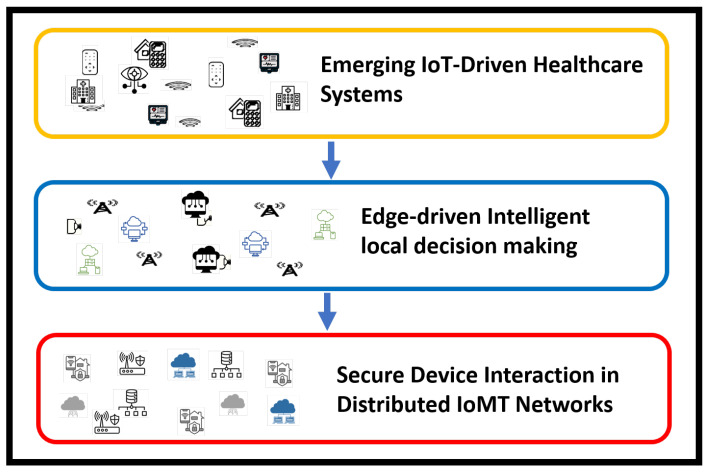
Phases of the proposed model for intelligent IoT communication with secure interfaces.

In this section, we present an AI-driven model that integrates security exploration via Support Vector Machines (SVMs) to improve the accuracy and resilience of innovative healthcare systems. The distributed network combines various electronic medical equipment, health sensors, and edge devices to ensure real-time data processing and secure information transmission. Figure 1 illustrates the architecture design of the proposed model, which comprises three main phases. Initially, IoT-based innovative systems are constructed to collect environmental data for analysis and processing. Secondly, edge-level local processing is performed to ensure the timely delivery and responsive communication. Ultimately, a secure interface is developed through trusted computation across devices, ensuring reliability in distributed systems. The significant components of the proposed methodology are discussed as follows.

### 3.1. System Architecture for Distributed IoMT Networks

Our system architecture is composed of health sensors $D=\{d_{1},d_{2},\ldots ,d_{n}\}$, edge nodes $E=\{e_{1},e_{2},\ldots ,e_{m}\}$ that perform local processing and reduce the additional consumption of resources of constraint devices. Equations (1) and (2) model the devices, edge nodes, and the cloud for optimized communication and decrease the network overhead.

(1) $$x_{i}\to e_{j}\;\forall d_{i}\in D,\forall e_{j}\in E$$

(2) $$e_{j}\to \mathrm{Cloud}\;\forall e_{j}\in E$$

The communication latency *L* between health devices and the cloud can be expressed using edge-level processing as given in Equation (3).

(3) $$L=\frac{D}{R}+T_{proc}$$

where data size is denoted by *D*, communication rate is denoted by *R*, and processing time at the edge or cloud is represented by $T_{proc}$. The objective function *F* as given in Equations (4) and (5) aims to minimize the communication latency and energy consumption in the IoMT system, with α as a weight factor to balance both terms. The model is subject to a constraint on the total data load in the network.

(4) $$\begin{array}{cc} F & =\sum_{i=1}^{n}\left(\frac{d_{i}\in D}{R}+T_{\mathrm{proc}}\right)+\alpha \sum_{i=1}^{n}E_{i}, \end{array}$$

(5) $$\begin{array}{cc} \mathrm{subject}\;\mathrm{to}\; & \sum_{i=1}^{n}d_{i}\leq C \end{array}$$

### 3.2. AI-Enabled Secure Edge System for Distributed Decision Making

In this section, the proposed model enhances the security of a distributed system by leveraging Support Vector Machines (SVMs) to detect network anomalies. Based on the selected features, the SVM classifier classifies IoMT traffic into normal or anomalous groups. The decision function of the SVM classifier is defined in Equation (6).

(6) $$f\left(x\right)=\mathrm{sign}\left(w^{T}x+b\right)$$

where f(x) is the output of the classifier, *x* is the input feature vector, *w* is the weight vector, and *b* is the bias term. Based on the selected features, the SVM classifier classifies IoMT traffic into normal or anomalous groups. The decision function of the SVM classifier is defined in Equation (7).

(7) $$f\left(x\right)=\mathrm{sign}\left(w_{1}\cdot x_{1}+w_{2}\cdot x_{2}+\ldots +w_{n}\cdot x_{n}+b\right)$$

where

- f(x) is the classifier output (normal or anomalous).
- $x=[x_{1},x_{2},\ldots ,x_{n}]$ is the feature vector, which includes:
  – packet size ($x_{1}$) and transmission rate ($x_{2}$);
  – response time ($x_{3}$) and data flow patterns ($x_{4}$);
  – and other network characteristics.
- $w_{i}$ is the weight associated with each feature $x_{i}$, and *b* is the bias term.

The final classification outcome is determined by the contribution of each feature, where the significance of each feature is captured by its corresponding weight $w_{i}$ in the detection of anomalies for a distributed IoMT system. The decision function, as shown in Equation (8), computes the weighted sum of the features $x_{i}$ along with the bias term *b*, and the classification is made based on the sign of the resulting value.

(8) $$f\left(x\right)=\mathrm{sign}\left(\sum_{i=1}^{n}w_{i}\cdot x_{i}+b\right)$$

Algorithm 1 governs the anomaly detection using an SVM classifier for an IoMT-based healthcare system.
**Algorithm 1:** Anomaly detection using SVM for IoMT traffic.

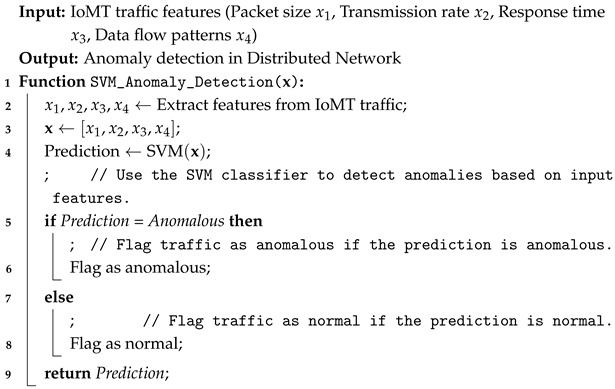


### 3.3. Integrating Edge Computing for Scalable IoMT System

In the next phase, edge computing is integrated into local devices to reduce latency and improve the efficacy of the proposed model. It improves system scalability by performing processing locally and reduces data transmission to the cloud. The decision to offload tasks is based on the optimization criterion, as defined in Equation (9):

(9) $$O\left(F\right)=\arg\min\left(L_{cloud}+E_{cloud},L_{edge}+E_{edge}\right)$$

where $L_{cloud},L_{edge}$ represent the latency for cloud and edge processing, respectively, and $E_{cloud},E_{edge}$ are the energy consumption for cloud and edge processing. Furthermore, to ensure accurate edge processing, Equation (10) defines the error function.

(10) $$E_{edge}=\frac{1}{n}\sum_{i=1}^{n}{\left(f\left(x_{i}\right)-y_{i}\right)}^{2}$$

where

- $E_{edge}$ is the error function measuring the performance of the edge node in processing the data.
- $f(x_{i})$ is the predicted output for the input feature $x_{i}$, and $y_{i}$ is the actual value.

Algorithm 2 presents a secure approach for task offloading and maintaining the authentic health services.
**Algorithm 2:** Edge computing for task offloading and error minimization.

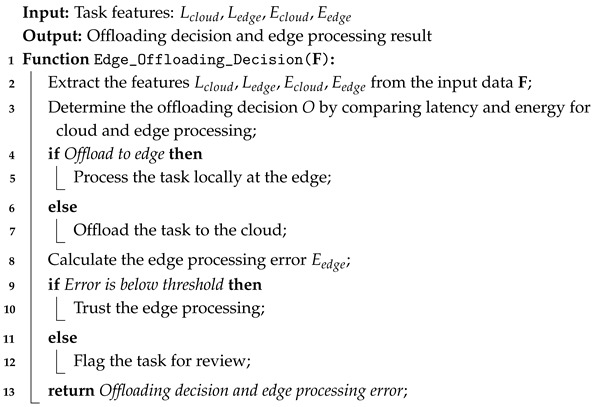


The proposed model achieves cyber resilience through system response and the timely detection of network threats by computing the resilience metric $R_{c}$. Equation (11) determines the detection time for *N* cyber attacks using the sum of individual detection times $T_{detection,i}$.

(11) $$T_{detection}=\sum_{i=1}^{N}\left(t_{detected,i}-t_{start,i}\right)$$

where

- $t_{detected,i}$ is the time of detection for the *i*-th attack.
- $t_{start,i}$ is the time the *i*-th attack starts.
- $t_{detected}$ is the time when the system identifies the attack.
- $t_{start}$ is the time when the attack begins.

On the other hand, the mitigation time $T_{mitigation}$ is the time taken to neutralize the attack and restore normal operations, as defined in Equation (12).

(12) $$T_{mitigation}=\max\left(t_{restored}-t_{detected}\right)$$

where

- $t_{restored}$ is the time when the system returns to normal operation after mitigation.
- $t_{detected}$ is the time when the attack is first detected.

Equation (13) defines the cyber resilience metric $R_{c}$, which is generalized to account for multiple attack events and their respective detection and mitigation times.

(13) $$R_{c}=\frac{\sum_{i=1}^{N_{a}}\left(T_{d}^{\left(i\right)}+T_{m}^{\left(i\right)}\right)}{T_{total}}$$

where

- $N_{a}$ is the number of detected attack events.
- $T_{d}^{\left(i\right)}$ is the detection time for the $i^{\mathrm{th}}$ attack.
- $T_{m}^{\left(i\right)}$ is the mitigation time for the $i^{\mathrm{th}}$ attack.
- $T_{total}$ is the system’s total operational time, including both normal and attack-response phases.

Algorithm 3 outlines the procedure for computing the cyber resilience metric $R_{c}$ in a distributed IoMT system.
**Algorithm 3:** Cyber resilience evaluation for distributed IoMT system.

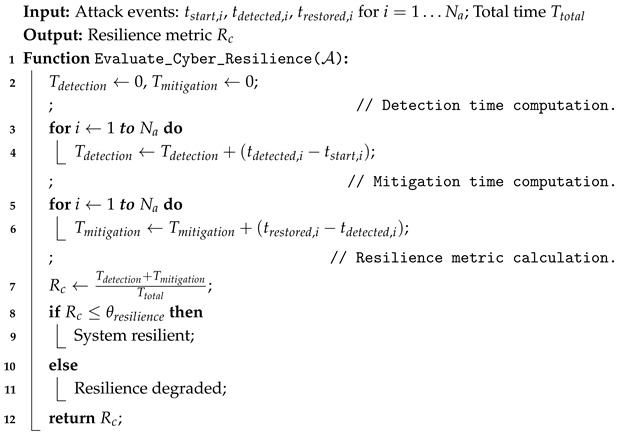


## 4. Simulation Configuration

In this section, we evaluate the proposed model and baseline solutions under controlled simulation scenarios. Simulations are performed in a 3000×3000 m area with IoT sensors and edge nodes. We vary the number of malicious nodes from 10 to 20 to assess the impact of privacy and security attacks on the system. Each configuration is executed 50 times independently and logged; the simulation round interval is 25 s. The simulation time per run is set to $T_{sim}=3000\;$. Table 2 summarizes the default simulation parameters used in the experiments.

### Discussion

**Figure 2:**
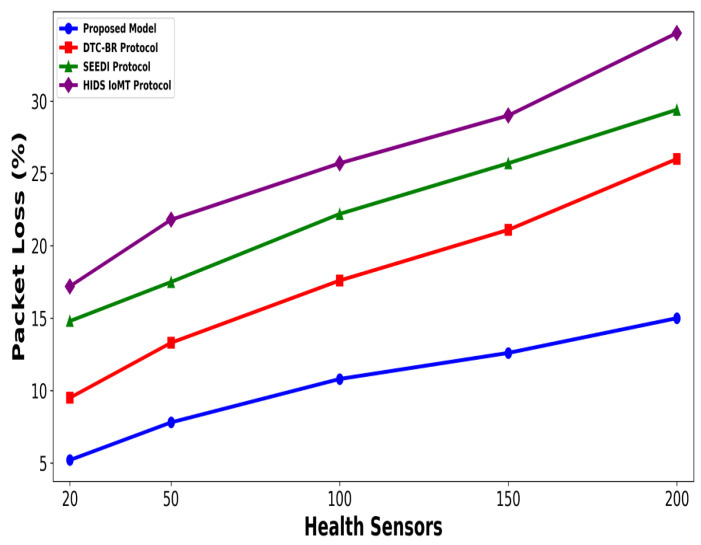
Performance of proposed model with DTC-BR Protocol, SEEDI Protocol, and HIDS-IoMT Protocol for packet drop ratio under varying health sensors.

**Figure 3:**
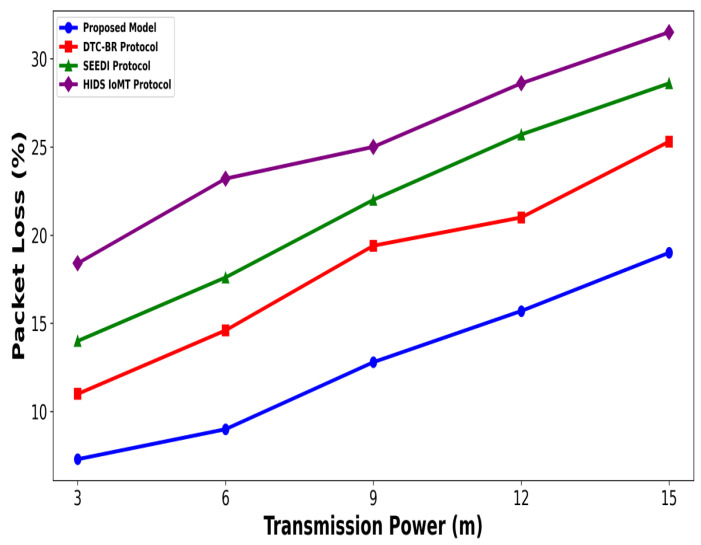
Performance of proposed model with DTC-BR Protocol, SEEDI Protocol, and HIDS-IoMT Protocol for packet drop ratio under varying transmission range.

**Figure 4:**
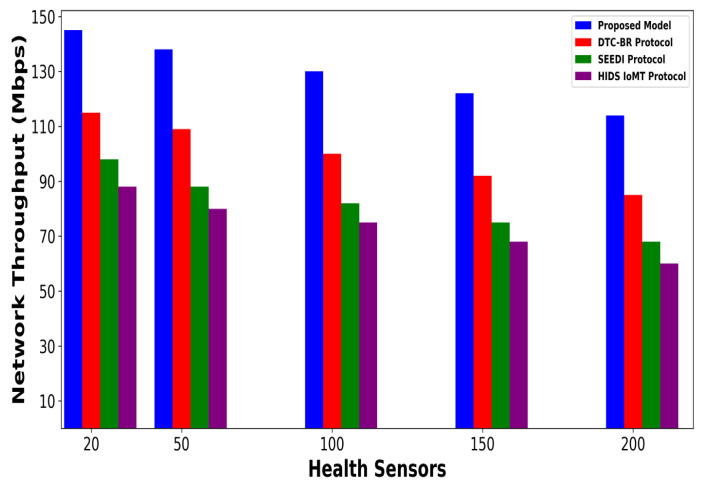
Performance of proposed model with DTC-BR Protocol, SEEDI Protocol, and HIDS-IoMT Protocol for network throughput under varying health sensors.

**Figure 5:**
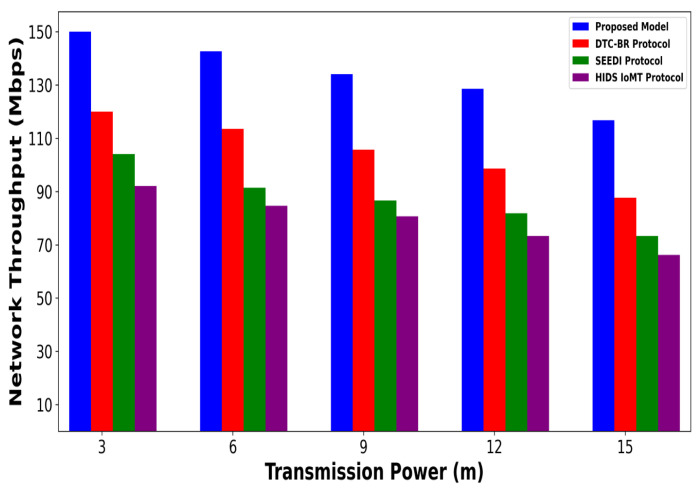
Performance of proposed model with DTC-BR Protocol, SEEDI Protocol, and HIDS-IoMT Protocol for network throughput under varying transmission range.

**Figure 6:**
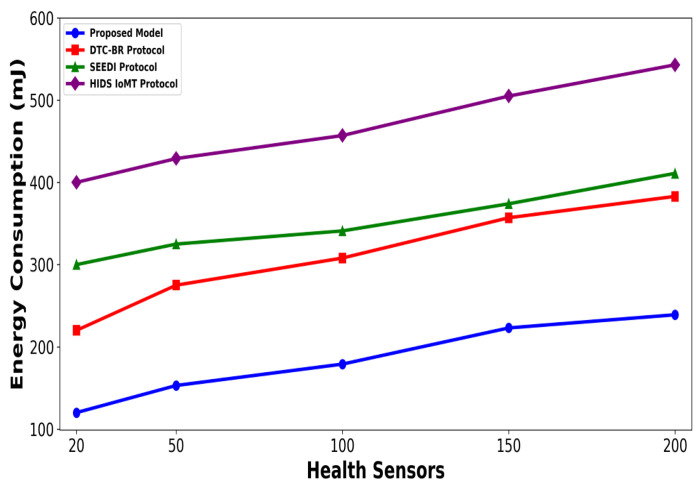
Performance of proposed model with DTC-BR Protocol, SEEDI Protocol, and HIDS-IoMT Protocol for energy consumption under varying health sensors.

**Figure 7:**
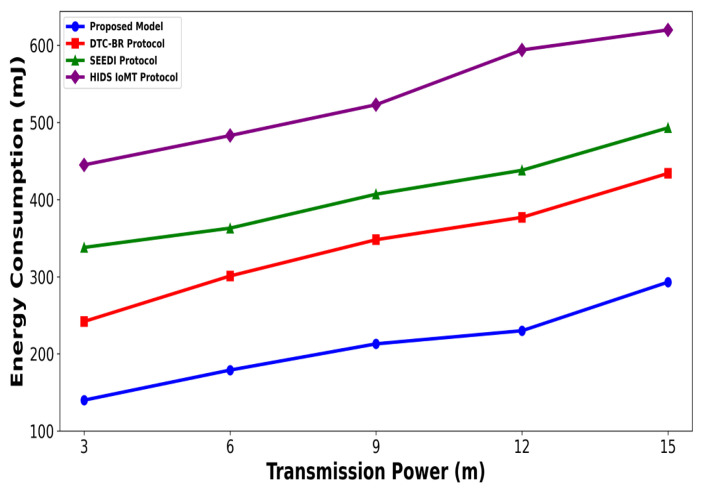
Performance of proposed model with DTC-BR Protocol, SEEDI Protocol, and HIDS-IoMT Protocol for energy consumption under varying transmission range.

**Figure 8:**
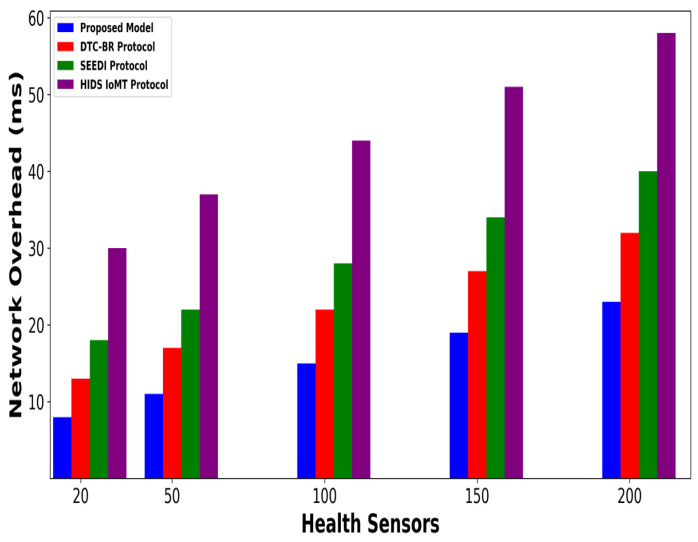
Performance of proposed model with DTC-BR Protocol, SEEDI Protocol, and HIDS-IoMT Protocol for network overhead under varying health sensors.

**Figure 9:**
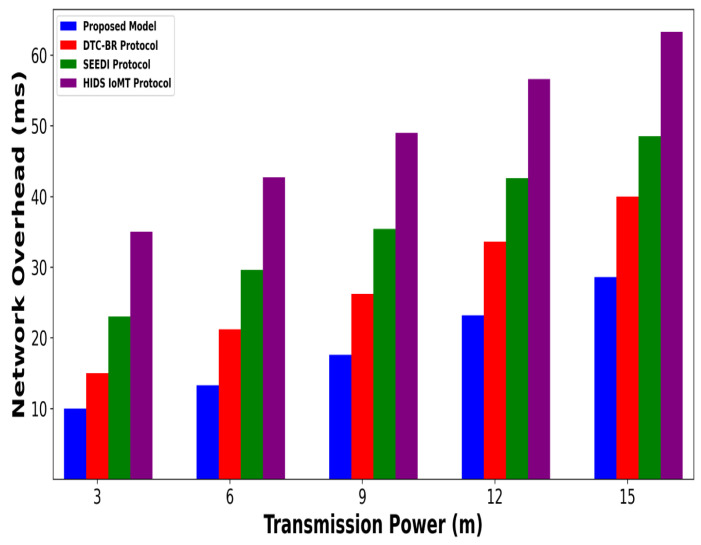
Performance of proposed model with DTC-BR Protocol, SEEDI Protocol, and HIDS-IoMT Protocol for network overhead under varying transmission range.

**Figure 10:**
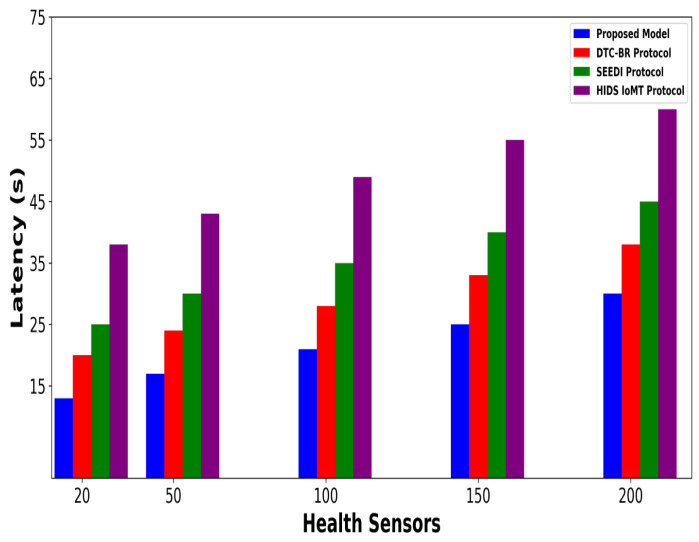
Performance of proposed model with DTC-BR Protocol, SEEDI Protocol, and HIDS-IoMT Protocol for latency under varying health sensors.

**Figure 11:**
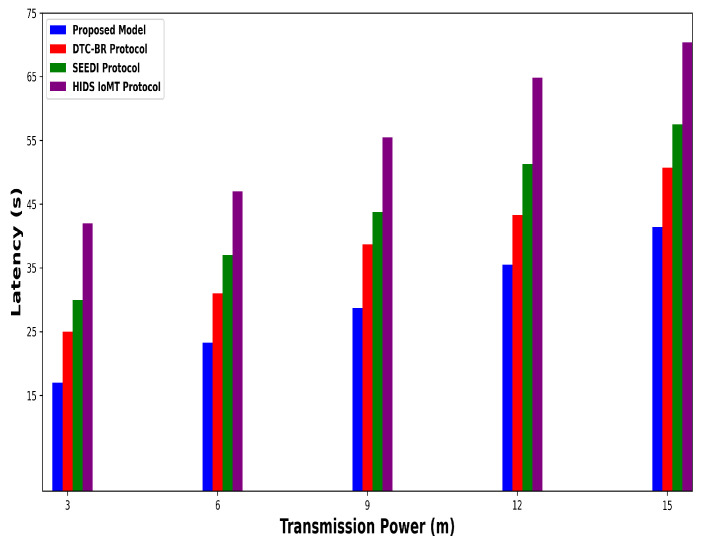
Performance of proposed model with DTC-BR Protocol, SEEDI Protocol, and HIDS-IoMT Protocol for latency under varying transmission range.

A significant improvement was noticed in terms of packet drop ratio by an average of 49% and 54% compared to existing methods, as shown in Figure 2 and Figure 3. This is made possible by utilizing edge-based computing and performing local processing with a low computational cost. A distributed decision-making approach is employed, utilizing SVM-based anomaly detection, while efficiently managing resources and optimizing the utilization of communication links. Moreover, the computation of cyber threats helps identify the most stable forwarding paths, thereby ensuring higher data reception at the destination. The security mechanisms embedded in the model prevent malicious devices from sending false route request packets, thereby reducing the risk of malicious traffic in the IoMT network. Its network throughput is compared with existing schemes in Figure 4 and Figure 5. The proposed model achieves average throughput improvements of 48% and 54% across varying sensor densities and transmission ranges, respectively. This improvement can be attributed to the integration of dynamic strategies that leverage distributed decision making and real-time data processing. The routing paths are optimized using a dynamic, weighted objective function that considers multiple feature extractions. Security policies are continuously updated via distributed evaluations, ensuring that unreliable or malicious nodes are excluded from the data-forwarding process. Figure 6 and Figure 7 illustrate the energy consumption performance of the proposed model and existing work under varying sensor densities and transmission ranges. Upon comparison, the proposed model demonstrates a significant improvement in energy efficiency, reducing the additional usage of network resources by an average of 50% and 57%, respectively. This enhancement is attributed to the integration of SVM classifier-based anomaly detection, AI-driven edge decision making, and real-time data processing. The devices are continuously monitored at the edge, and an SVM classifier identifies potential cyber threats based on abnormal data patterns or malicious behavior. When such threats are detected, the system reformulates the communication routes by exploring surrounding environmental conditions and adjusting node parameters. Also, the edge nodes are responsible for validating the identities of connected devices before forwarding data to the cloud. Figure 8 and Figure 9 compare the proposed model to existing approaches in terms of network overhead. Node overhead is a critical consideration when designing and implementing IoT-enabled distributed networks, especially in resource-constrained environments. The experimental analysis demonstrates that the proposed model reduces node overhead by an average of 44% and 52% under varying sensor densities and transmission ranges. This improvement is attributed to learning-based decision making, which involves exploring various feature extractions of the device under dynamic conditions. This approach uses distributed, real-time decision-making algorithms to optimize communication and resource management at the edge. In Figure 10 and Figure 11, the performance analysis of the proposed model is compared for latency over varying transmission ranges and health sensors. The results analysis reveals an improvement of the proposed model by an average of 42 and 50% and 49%, respectively. This is made possible through the exploration of the SVM algorithm for network intelligence and communication detection, utilizing key and dynamic features such as response time, data size, and data flow patterns, in healthcare applications. It timely detects unauthorized access in the system and reduces abnormal device behavior, ultimately improving the efficient delivery of health records. In addition, resources are utilized in a balanced manner, and their consumption is reduced to tackle network congestion and mitigate overloaded routes for the transmission of crucial e-health data.

## 5. Conclusions

Smart applications with edge computing are interconnected using emerging technologies to sense and process the collected data. It timely provides the request to remote devices and facilitates advancing of the healthcare system. Many approaches have been proposed to address the timely delivery of patients’ data to cloud systems, enabling the analysis of their condition and the personalization of treatments by healthcare providers. Although distributed computing across heterogeneous systems is one of the most challenging research problems addressed by most existing approaches, resource optimization with adaptive healthcare routing remains a pressing issue due to the limited processing and storage capabilities of IoMT networks. Moreover, securing the sensitive data against cyber threats without additional energy consumption is also a significant issue. We proposed an AI-enabled edge computing model for the timely detection of malicious threats in distributed healthcare applications, thereby enhancing the quality of service (QoS) for intermediate devices. The trust is also incorporated in an unpredictable environment, providing a more reliable communication paradigm that supports efficient system operations. Our future plan is to test the model with realistic collected data and integrate a centralized controller to enhance network segmentation and adjust the security measures against massive network traffic. 

## Figures and Tables

**Table 1:** Summary of research contributions and limitations.

Existing Approaches	Contributions	Limitations
DTC-BR Protocol [28]	Enhances MWSN performance using dual-tier clustering and virtual network zones. The protocol divides the network into two zones: the main connectivity zone (MCZ) and the candidate cluster zone (CCZ), thereby improving energy efficiency and scalability.	May not be effective in highly dynamic environments with frequent topology changes.
FFAO [29]	Integrates multi-objective optimization criteria to strengthen the network stability and reliability. It improved the routing process while selecting the optimal path for transmitting IoT data.	May face additional challenges such as overheads, and network delay in coping with resource optimization.
QoS-aware Routing Strategy [30]	Proposes a software architecture for data collection and communication in IoT-enabled smart applications, utilizing Chaotic Bird Swarm Optimization (CBSO) for cluster formation and Improved Differential Search (IDS) to assess node reliability.	High computational complexity during the data collection phase may result in increased overhead for real-time applications.
SEEDI Protocol [31]	Presents a sink-mobility-based energy-efficient data dissemination protocol for IoMT, designed to address the hot-spot problem in static WBANs. It deploys energy-powered nodes at the periphery to collect patient data and uses the Remora Optimization Algorithm (ROA) to select cluster heads.	Faces challenges in highly dynamic environments with fluctuating traffic patterns, which may hinder real-time data transmission.
HIDS-IoMT Protocol [32]	Introduces a hybrid deep learning-based intrusion detection system (HIDS-IoMT) combining CNN for feature extraction and LSTM for sequence prediction, implemented on a Raspberry Pi with fog computing for decentralized processing.	High computational demands of deep learning models may result in slow performance on resource-constrained devices in real-time deployments.
HSPBCI framework [33]	Improves the security level for healthcare system with blockchain network and effective key management for authorized data access.	Limited in network scalability and enhanced data latency while processing high amount of health records.

**Table 2:** Simulation parameters.

Parameter	Value/Description
Simulation area	3000×3000m
Simulation duration	3000 s
Number of executions	50 independent runs
Round interval	25 s
Sensor nodes	20, 50, 100, 150, 200
Edge nodes	15
Sink nodes	3
Malicious nodes	5–15
Mobility model	Random waypoint
Packet size	128–1024 bytes
Transmission power	3–15 m
Initial energy	5000 mJ

## Data Availability

All data is available in the manuscript.
